# Male Osteoporosis in the Elderly

**DOI:** 10.1155/2015/907689

**Published:** 2015-09-20

**Authors:** Patrizia D'Amelio, Giovanni Carlo Isaia

**Affiliations:** Department of Medical Science, University of Torino, 10126 Torino, Italy

## Abstract

Osteoporosis is now recognized as an important public health problem in elderly men as fragility fractures are complicated by increased morbidity, mortality, and social costs. This review comprises an overview of recent findings in pathophysiology, diagnosis, and treatment of male osteoporosis, with particular regard to the old population.

## 1. Introduction

Osteoporosis is a systemic skeletal disease characterized by reduced bone density and microarchitectural deterioration of bone; this leads to increased fracture risk [1].

Bone fractures are a major health problem in the increasingly older population. The old suffering from femoral fractures will die within a year (15–25%) or become dependent (50%) [2, 3]. A single vertebral fracture doubles the risk of subsequent femoral fracture within a year and multiple vertebral fractures impair patients' quality of life and increase mortality [4]. Major osteoporotic fractures at wrist, spine, and hip are a social and economic burden; in developed countries, the lifetime risk for osteoporotic fractures at the wrist, hip, or spine is 30% to 40%, very close to that for coronary heart disease [5, 6].

Although osteoporosis is perceived by the general population as a women disease, 1 in 8 men aged older than 50 years will experience a fragility fracture during his lifetime; the most common sites for fragility fractures in men are forearm, vertebrae, and hip, but also fractures of other sites as ribs, pelvis, and clavicle are associated with male osteoporosis [7–9].

Almost 30% of hip fractures occur in men [10] and mortality, within the first year after a hip fracture, is higher in men compared to women [11, 12]; compared to women, men suffering from femoral fractures have 2- to 3-fold increased mortality risk [11]; the reason for this gender difference is unknown.

Men do not experience rapid bone loss as women do after menopause [13]; instead; they undergo a slow bone loss with age [14]; this bone loss begins by the sixth decade at an average rate of 0.5% to 1.0% per year and is accompanied by growing incidence of fractures [15].

Considering these data, osteoporosis in old men should be considered as a serious public health concern and as a life threatening disease; despite this consideration, male osteoporosis is still an underdiagnosed and undertreated condition. Thus, the aim of this paper is to review the current knowledge on the pathophysiology, diagnosis, and treatment of osteoporosis in old men.

## 2. Pathophysiology of Male Osteoporosis in the Elderly

Bone is a living tissue that undergoes continuous remodeling due to the combined action of bone cells: the osteoblasts (OBs) that build up new bone matrix and the osteoclasts (OCs) that resorb bone. Within the bone matrix, osteocytes (OSs), the mature form of OBs, regulate bone turnover by directing OBs and OCs activities. In osteoporosis, OBs and OCs activities are unbalanced with increased bone resorption and decreased bone deposition; this imbalance turns in bone loss and increased fracture risk.

Several diseases alter the balance between bone formation and bone resorption and induce bone loss; in women, 20 to 40% of osteoporosis is secondary to extraskeletal diseases, and this percentage raises until 65% in men [16, 17].

Other than secondary causes, aging is a primary cause of bone loss in men as well as in women; it induces bone loss through hormonal changes and age-related osteoblast dysfunction.

### 2.1. Hormonal Changes during Aging

Hormonal changes during aging are responsible for bone loss; in particular, decreased levels of sexual steroid and relative increase in cortisol negatively influence bone remodeling.

It is widely accepted that the decrease in sex steroid concentrations with age is associated with decreased bone density and increased fracture risk in men [18–20]; nevertheless, the decline of testosterone in men is gradual and not common to all the aged population. The decrease in bioavailable estradiol more than in testosterone appears to be the cause of bone loss in old men. A recent paper on the wide cohort of men participating in the MrOs study demonstrates that men with the lowest bioavailable estradiol had greater risk of fractures, whereas men with the lowest free testosterone had no increased fracture risk after adjustment for estradiol [21]. Thus, the authors suggest that the bioavailability of estradiol, more than testosterone, is responsible for increased fracture risk in old men.

Excess of glucocorticoids both endogenous and exogenous is known to be detrimental for bone; glucocorticoids affect bone mainly by decreasing OB function [22]. Glucocorticoid action is dependent upon the expression of 11 beta-hydroxysteroid dehydrogenase isozymes, which interconvert active cortisol and inactive cortisone. Bone tissue is able to convert cortisone in active cortisol thanks to this enzyme, whose expression increases with aging [23]. Thus, old persons are more sensible to endogenous and exogenous glucocorticoid; this results in a relative hypercortisolism and possibly in bone damage.

### 2.2. Age-Related Osteoblast Dysfunction

In old persons, OBs function is reduced with a consequent decrease in bone formation; processes involved in this mechanism have been studied with controversial results; age-related changes in OBs recruitment, differentiation, and function have been analyzed.

OBs derive from the differentiation of skeletal mesenchymal stem cells (MSC). The ancestral MSC is able to differentiate in vitro into OBs, adipocytes, or chondrocytes [24] and to self-renew [25]. It has been suggested that a reduced ability of MSC to differentiate into OBs may play a role in aging-related bone loss; however, studies on humans show controversial results. Some studies show age-related decrease in the number of MSCs able to differentiate into OBs [26–28] whereas others do not [29–32]. Thus, it is reasonable to hypothesize that age-related changes in number of MSCs able to differentiate into OBs do not play an essential role in the pathophysiology of male osteoporosis in the old.

The ability of MSC to differentiate into OBs has also been studied and a recent work done in mice suggests that age impairs this ability [33, 34]. Thus, this could be one of the mechanisms explaining reduction in bone formation with age.

Moreover, OBs may modify their environment by acquiring a typical senescent secretory phenotype involving inflammatory cytokines, growth factors, and proteases [35, 36], thus contributing to increased OCs activity and bone loss.

### 2.3. Vitamin D Deficiency

It is well known that vitamin D plays an important role in regulating calcium metabolism and that its deficiency leads to bone demineralization and increased fracture risk [37].

More than 80% of vitamin D derives from cutaneous synthesis whereas only 20% comes from diet; cholecalciferol is converted into its active form 1,25-dihydroxyvitamin D3 [1,25(OH)D3] by two hydroxylations in the liver and in the kidney. Kidney cells hydroxylate vitamin D thanks to the enzyme 1 alpha hydroxylase that is under PTH control.

1,25(OH)D3 binds its nuclear receptor (VDR) and contributes to calcium and phosphorus homeostasis; in the small intestinal cells, the activation of VDR increases calcium absorption and maintains appropriate calcium levels thus improving bone mineralization [38].

If the calcium intake is reduced, PTH rises and more vitamin D is converted into 1,25(OH)D3; this active form of vitamin D increases calcium level by stimulating OCs activity, thus increasing bone resorption with calcium and phosphorus release in the blood stream [38, 39].

There is no global consensus on the appropriate cut-off for the definition of hypovitaminosis D: serum 25(OH)D ranging from <25 to <75 nmol/L has been used in defining hypovitaminosis D [40, 41]. This lack of consensus results in different prevalence of hypovitaminosis D ranging from 1 to 80% [42–44]. Despite these observations, it is known that hypovitaminosis D is largely prevalent among adult population of both genders and that the incidence of hypovitaminosis D increases with age due to changes in lifestyle but also to decreased cutaneous synthesis [45].

For the above mentioned reasons, hypovitaminosis D has to be considered in the diagnostic processes of male osteoporosis in the elderly and a correct vitamin D supplementation has to be guaranteed in order to ensure maximum benefit of treatment.

## 3. Diagnosis of Male Osteoporosis

As well as in women, diagnosis of osteoporosis in men is based on the measurement of bone mineral density (BMD). Based on BMD measurement, the criteria used for the diagnosis are established by the World Health Organization (WHO). The WHO has defined as osteoporotic the patients whose BMD is lower than −2.5 standard deviations (*T*-score) with respect to reference population and as affected by severe osteoporosis the patients with the same BMD threshold and the presence of one or more fragility fractures [46].

In order to avoid biases due to individual, skeletal site and technique variation, WHO has recommended the use of a unique reference population (the NHANES III, women aged 20–29 years), the femoral neck as site for diagnosis, and a single technology: the Double Emission X-ray Absorptiometry (DXA) [47–49]. Thus, the diagnosis of osteoporosis in men is achieved by comparing the measured BMD to the BMD of a women reference population. Wide meta-analyses on more than 39,000 primary data confirmed this assumption, showing that BMD referred to the NHANES III population predicts fracture risk in men as well as in women [50].

The guidelines of the Endocrine Society [51] and of Osteoporosis Canada [52] recommend the use of spine and femur for the diagnosis of osteoporosis, but allow the use of distal radius when spine or hip scans cannot be interpreted and for men with hyperparathyroidism or receiving androgen deprivation therapy for prostate cancer.

BMD alone does not account for the whole bone mechanical properties [53, 54]; thus, several approaches have been performed to identify parameters of bone quality to be used in clinical evaluation of male osteoporosis. These approaches are based on the observation of gender related differences in bone architecture as differences in bone size [55]; also the aging process induces changes in bone morphology that may account for increased fracture risk, as decreased osteon size [56–58], and increased diameter of the Haversian canals [56].

Recent studies also support a role for intracortical porosity and its distribution in determining age-related fracture risk and gender differences. In particular, the increased cortical porosity in old men may be one of the determinants of increased fracture risk [59, 60].

Despite this good evidence for sex-specific and age-related differences in bone quality, the WHO and the International Osteoporosis Foundation do not recommend the clinical evaluation of these parameters in clinical fracture risk assessment in male osteoporosis [49].

The evaluation of clinical risk factors for low BMD may help identifying patients to be screened with DXA. Several risk factors have been associated in large studies with increased risk fractures in men as low body mass index, increasing age, history of prior fracture, hypogonadism, stroke, excessive alcohol consumption, current smoking, long-term corticosteroid use, recent falls, impaired neuromuscular function, and diabetes [61, 62].

After identifying patients with low BMD, it is necessary to exclude secondary osteoporosis before treatment. Several conditions cause secondary osteoporosis, summarized as follows:

*The Main Causes of Secondary Osteoporosis in Men (Data from [53, 63])*

 Alcoholism.
 Chronic obstructive pulmonary disease.
 Gastrointestinal disorders:
   malabsorption syndromes,
   inflammatory bowel disease, celiac sprue,
   primary biliary cirrhosis,
   gastrectomy.
 Poor nutrition:
   low serum levels of vitamin D,
   low calcium.
 Hypercalciuria.
 Endocrine disorders:
   hyperthyroidism,
   hyperparathyroidism,
   hypercortisolism.
 Hypogonadism:
   idiopathic,
   androgen deprivation therapy for prostate cancer,
 Drug induced:
   anticonvulsants,
   chemotherapeutics,
   glucocorticoids,
   thyroid hormone.
 Neuromuscular disorders.
 Posttransplant osteoporosis.
 Mastocytosis.
 Malignancies.
 Rheumatoid arthritis.
 Autoimmune diseases other than rheumatoid arthritis.

A more complete review of causes of secondary osteoporosis in men is beyond the scope of this review. Accordingly, the reader is referred to two recently published papers for extensive review [62, 64].

## 4. Treatment

The goal for treatment of male osteoporosis is to reduce the fracture risk; however, the majority of clinical trials with fractures prevention as primary end point have been performed in women. Study in men typically addresses surrogate end points as changes in BMD and in markers of bone turnover, the use of surrogate markers instead of fracture reduction is justified on the basis of therapeutic equivalence; the BMD changes observed in men are comparable to those observed in women; thus, it is assumed that efficacy in fractures reduction will also be similar [49].

Before reviewing evidence on treatment efficacy, it appears important to focus on whom to be treated.

### 4.1. Whom to Treat?

BMD is not the only determinant of fracture risk, as it has been demonstrated that in male patients experiencing nonvertebral fractures only 21% has a* T*-score below −2.5 [8]. Thus, suggesting that BMD* T-*score cannot be used as a threshold to decide treatment, it is necessary to consider risk factors other than BMD to decide whom to treat.

To this end, an algorithm that integrates clinical risk factors with BMD measurement has been developed by the WHO. The algorithm called fracture risk assessment tool (FRAX) estimates the 10-year probability of a hip or major osteoporotic fracture in men and women [64]. FRAX considers age, sex, weight, height, previous fracture, parental history of hip fracture, current smoking, secondary osteoporosis, glucocorticoid exposure, rheumatoid arthritis, consumption of three or more units of alcohol per day, and BMD measured by DXA at the femoral neck, if available, to estimate fracture risk. Different FRAX models can be used according to different country-specific epidemiology of fractures. FRAX is freely available at http://www.shef.ac.uk/FRAX/index.aspx.

Other fracture risk assessment tools have been proposed [52, 65, 66]; the performance of these tools has not been extensively studied in a male population.

Treatment guidelines for male osteoporosis differ in different countries; the presence of a fragility fracture due to osteoporosis as indication to treatment is common amongst different guidelines. Guidelines for treatment of male osteoporosis from National Osteoporosis Foundation [67], Endocrine Society [51], and Osteoporosis Canada [52] are summarized in Table 1.

### 4.2. Which Treatment?

Together with pharmacological treatment, it is appropriate to ensure adequate calcium and vitamin D intake and regular physical activity with weight-bearing exercises and avoid alcohol intake and tobacco use. All the guidelines include the recommendation for nonpharmacological treatment [48, 51, 52]. Strategies of fall prevention are suggested by NOF and Osteoporosis Canada [48, 52].

There is some evidence to suggest an active role for calcium and vitamin D supplementation in preventing fractures in old men [68], whereas physical exercise may have a more major role in reducing the risk of falls [69], than in increasing BMD.

*Alendronate* [70],* risedronate*, [71], and* zoledronic acid* [72] have been shown to reduce the risk of vertebral fragility fractures in men and are approved by FDA. Among these, only risedronate has been proven to be efficacious also in the reductions of nonvertebral [73] and hip [74] fractures in men. Bisphosphonates are proven to be effective also in male osteoporosis secondary to glucocorticoid [75] or androgen deprivation therapy [63, 76, 77].

*Denosumab* has recently been approved by FDA for the treatment of male osteoporosis; FDA approved the new indication after examining data from the ADAMO trial. ADAMO trial demonstrated that, in men with low BMD, denosumab treatment increases BMD and reduces bone resorption regardless of baseline testosterone levels, BMD status, estimated fracture risk, or age [78]. Denosumab is also approved to increase bone mass in patients receiving androgen deprivation therapy for nonmetastatic prostate cancer [79].

Treatment with daily subcutaneous injection of 1–34 parathyroid hormone (*Teriparatide*) has been approved by FDA for the treatment of primary osteoporosis in male [80] as well as of glucocorticoid induced osteoporosis [81].

In Europe,* Strontium Ranelate* is also approved for the treatment of male osteoporosis; the increase in lumbar spine and hip BMD in men with osteoporosis obtained with this drug is similar to the one obtained in women [82].

As it is known, hypogonadism is accompanied by increased bone turnover and increased fracture risk [83, 84]; thus it seems interesting to evaluate the effect of* testosterone* treatment in male osteoporosis. Some observational studies suggest that treatment with testosterone reduces bone turnover and increases BMD, whereas effect on fracture risk has not been assessed [84, 85].

Nevertheless, due to adverse effects on hematocrit, prostate, and cardiovascular system and to the lack of data on fracture prevention, testosterone replacement therapy, despite being indicated in symptomatic hypogonadism, is not indicated for the treatment of male osteoporosis. Thus, in patients with symptomatic hypogonadism and osteoporosis, a treatment other than testosterone to prevent fractures is recommended [49].

### 4.3. Future of Treatment: Some Perspectives

New treatments are under development; in particular, anti-sclerostin antibodies, odanacatib (a cathepsin-K inhibitor), and abaloparatide (PTH-related peptide analog) are in advanced stages of clinical evaluation, whereas others as dickkopf-1 antagonists and calcilytics are in earlier clinical and preclinical phases of development.

Anti-sclerostin antibodies increase bone formation by inhibiting sclerostin (SOST). SOST is produced mainly by osteocytes and inhibits the Wnt pathway; inhibition of Wnt pathway decreases OBs formation and activity and consequently decreases bone formation. Inhibiting SOS by an antibody increases OBs activity. Currently, there are two anti-sclerostin monoclonal antibodies under study,* Romosozumab* [86] and* Blosozumab* [87]. Both the antibodies are safe and effective in increasing BMD in postmenopausal women; moreover, both drugs were effective in increasing markers of bone formation and reducing markers of bone resorption; nevertheless, Romosozumab is in a more advanced phase of development.

Romosozumab has similar effects both in men and women with low bone mass [88], whereas there are no studies for Blosozumab in men. Phase 3 trial with Romosozumab in men with osteoporosis (clinical trials.gov: NCT02186171) is ongoing, whereas there is no ongoing trial for Blosozumab in male osteoporosis.

*Odanacatib* specifically targets the protease cathepsin-K, which has a role in OCs resorbing activity; in particular, this protein is released by OCs and degrades bone matrix proteins. Odanacatib reduces bone resorption without affecting OCs number and hence preserves bone formation [89]. In a phase III trial, odanacatib 50 mg once weekly significantly increases BMD and ameliorates bone structure as compared to placebo in postmenopausal women [90]. A randomized, placebo-controlled phase III trial on Odanacatib in osteoporotic men is ongoing (clinical trials.gov: NCT01120600).

*Abaloparatide* is a PTH-related peptide analog that acts as an anabolic agent and increases OBs activity. Results of phase II trial show that abaloparatide is effective in increasing BMD at both lumbar spine and femoral neck [91]. Currently, there are no studies for abaloparatide in men with osteoporosis.

Other drugs targeting the Wnt pathway inhibitors and activators are currently in early stage of development; the most promising seems to be the antibody against dickkopf-1 (Dkk-1). Preclinical findings suggest that the inhibition of DKK-1 action has a bone anabolic effect [92]; nevertheless, there are no human data confirming these findings. The phase I trial of an anti-DDK-1 antibody (RN546) registered as clinical trial NCT01293487 was completed, but currently the results have not been published.

Other potential candidates as anabolic treatment in osteoporosis are calcilytic agents; these drugs act as calcium-sensing receptor antagonists (e.g., ronacaleret, JTT-305/MK-5442, and AXT914) and stimulate endogenous pulsatile PTH secretion. Despite some promising animal data, up to now, calcilytic drugs did not show bone anabolic activity [93, 94].

## 5. Conclusions

Although the incidence of fractures in men is increasing due to the ageing population, male osteoporosis remains an underdiagnosed and undertreated disease. Nevertheless, there is growing awareness of the problem as a significant public health concern.

Osteoporotic fractures increase morbidity and mortality of old men together with a considerable increase in public health costs.

The pathophysiology of primary male osteoporosis comprises hormonal changes and cellular ageing. More than 65% of fractures in men are due to secondary osteoporosis.

Clinical guidelines to address osteoporosis treatment in men comprehend the diagnosis of fragility fractures, the measurement of BMD, and fracture risk estimation mainly through FRAX.

The approved pharmacological treatments have shown to be effective in male osteoporosis; nevertheless, more research is needed to address the efficacy in preventing nonvertebral fractures and to examine the comparative effectiveness of the various osteoporosis treatments in male.

## Figures and Tables

**Table 1:** Summary of treatment guidelines for male osteoporosis.

Organization	Pharmacological treatment recommendations
National Osteoporosis Foundation (NOF) [48]	(i) Age ≥ 50 (ii) Hip or vertebral fracture (iii) *T*-score less than −2.5 at femoral neck, total hip, or lumbar spine; *T*-score between −1.0 and −2.5 at the femoral neck or lumbar spine (iv) 10-year probability of a hip fracture >3% or a 10-year probability of a major fracture >20% based on the US-adapted FRAX

The Endocrine Society [51]	(i) Hip or vertebral fracture without major trauma; BMD of spine, femoral neck, or total hip 2.5 SD or more below mean of normal young males (ii) *T*-score between −1.0 and −2.5 at the femoral neck or lumbar spine plus a 10-year probability of a hip fracture >3% or a 10-year probability of a major fracture >20% based on FRAX (iii) Age ≥ 50 and long-term glucocorticoid therapy (equivalent to 7.5 mg or greater of prednisone for 3 months)

Osteoporosis Canada [52]	(i) 10-year fracture risk >20% assessed with CAROC^*∗*^ or Canadian FRAX (ii) Prior hip or spine fracture, or multiple prior fractures (iii) Moderate 10-year fracture risk (between 10% and 20%), if there are the following risk factors: vertebral fracture identified by imaging, previous wrist fracture in those over the age of 65 years or with *T*-score less than or equal to −2.5, lumbar spine *T*-score much smaller than femoral neck *T*-score, androgen deprivation therapy for prostate cancer, long-term glucocorticoid use, recurrent falls, or other disorders associated with osteoporosis, bone, loss, or fractures

^*∗*^CAROC: Canadian Association of Radiologists and Osteoporosis.
