# Corrigendum to “Male Osteoporosis in the Elderly”

**DOI:** 10.1155/2017/9839017

**Published:** 2017-07-02

**Authors:** Patrizia D'Amelio, Giovanni Carlo Isaia

**Affiliations:** Department of Medical Science, University of Torino, 10126 Torino, Italy

In the article titled “Male Osteoporosis in the Elderly” [1], there was an error regarding the FRAX® tool, which should be clarified as follows:

The article notes: “To this end, an algorithm that integrates clinical risk factors with BMD measurement has been developed by the WHO. The algorithm called fracture risk assessment tool (FRAX).” However, the World Health Organization (WHO) did not develop, test, or endorse the FRAX tool or its recommendations [2]. The metabolic bone disease unit at the University of Sheffield that developed FRAX was a WHO Collaborating Centre from 1991 to 2010, but treatment guidelines must undergo a formal process before they can be endorsed by the WHO.

## References

[1] D’Amelio P., Isaia G. C. (2015). Male Osteoporosis in the Elderly. *International Journal of Endocrinology*.

[2] Ford N., Norris S. L., Hill S. R. (2016). Clarifying WHO’s position on the FRAX® tool for fracture prediction. *Bulletin of the World Health Organization*.

